# The Multidisciplinary Mobile Unit (MMU) Program Bringing Hospital Specialist Geriatric Competencies at Home: A Feasible Alternative to Admission in Older Patients with Urgent Complaints

**DOI:** 10.3390/jcm13092720

**Published:** 2024-05-06

**Authors:** Antonio Nouvenne, Andrea Ticinesi, Carmine Siniscalchi, Martina Rendo, Nicoletta Cerundolo, Alberto Parise, Giampiero Castaldo, Giulia Chiussi, Richard Carrassi, Angela Guerra, Tiziana Meschi

**Affiliations:** 1 Department of Medicine and Surgery, University of Parma, Via Antonio Gramsci 14, 43126 Parma, Italy; antonio.nouvenne@unipr.it (A.N.); andrea.ticinesi@unipr.it (A.T.); richard.carrassi@gmail.com (R.C.); angela.guerra@unipr.it (A.G.); tiziana.meschi@unipr.it (T.M.); 2 Parma University Hospital—Azienda Ospedaliero-Universitaria di Parma, Via Antonio Gramsci 14, 43126 Parma, Italy; ncerundolo@ao.pr.it (N.C.); aparise@ao.pr.it (A.P.); castaldog@ao.pr.it (G.C.); gchiussi@ao.pr.it (G.C.); 3 Primary Care Department, Parma District, Azienda Unità Sanitaria Locale di Parma, Strada del Quartiere 2/A, 43125 Parma, Italy; mrendo@ausl.pr.it

**Keywords:** hospital at home, lung ultrasound, geriatric assessment, geriatric emergency medicine

## Abstract

**Background/Objectives**: Older patients are subject to a high number of Emergency Department (ED) visits and hospitalizations. Innovative strategies to manage geriatric urgencies in the community are thus needed. **Methods**: In this prospective observational study, we examined the case mix of a hospital-based domiciliary urgent care service tailored to older patients, called Multidisciplinary Mobile Unit (MMU), from January to September 2023. The service, activated by general practitioners or territorial specialists during workdays, provided domiciliary geriatric assessment, point-of-care diagnostics, including multi-site ultrasound and lab tests, and therapeutical measures, including intravenous treatment and insertion of invasive devices, with the goal of reaching on-site stabilization and avoiding ED referral. We collected data regarding multimorbidity, polypharmacy, and frailty according to the Clinical Frailty Scale (CFS), reasons for MMU activation, and diagnostic and therapeutical services provided. The assessed outcomes were immediate hospitalization after a visit, 30-day admission, and 30-day mortality. **Results**: Participants (n = 205, 102 M) were mostly aged (median age 83 years old), with multimorbidity and frailty (CFS median 6). The most frequent reasons for MMU activation were dyspnea (49%), cough (34%), and musculoskeletal pain (17%), while the commonest diagnostic test provided was thoracic ultrasound (81%). Only five patients (2.4%) were hospitalized immediately after MMU visit. The 30-day rate of hospitalization was 10.2%, with age, cancer, and abdominal pain as independent predictors on a stepwise binary logistic regression model. 30-day mortality was 4.9%. **Conclusions**: The MMU model is a feasible strategy to manage geriatric urgencies, especially involving the cardiorespiratory system, is associated with good outcomes and may prevent ED visits.

## 1. Introduction

Older patients with frailty and multimorbidity account for a substantial fraction of Emergency Department (ED) visits worldwide [1]. EDs, however, are hardly ever structured to effectively manage the multidimensional complexity of geriatric patients [2,3], in whom acute diseases or exacerbations of chronic illnesses often exhibit atypical presentations and overlapping clinical pictures [4]. This may lead to diagnostic delays, long ED boarding times, inappropriate hospitalizations, and, ultimately, poor outcomes, including increased risk of delirium and hospital-related infection, loss of independence, and mortality [2,5,6]. 

Advanced healthcare systems have elaborated several strategies to avoid hospitalizations in older patients [2,7]. In some cases, older patients may be unnecessarily referred to EDs for exacerbations of ambulatory-sensitive conditions, for which the existence of tailored community care pathways may represent a valid alternative to ward admission [8]. Community care programs, however, may also be structured to manage higher levels of urgency and clinical complexity, offering high home care standards in cases where hospitalization should be normally recommended [2,7].

Hospital-at-home programs are acute clinical services taking staff, equipment, technologies, therapies, and skills, usually provided in hospital, directly at the patient’s own home [9,10,11,12,13]. These services generally substitute inpatient care after an initial diagnostic evaluation, which can be either performed in community medical services or in the hospital [9,10,11]. Such programs generally include administration of therapeutical measures, remote monitoring, periodical clinical follow-up, and nurse-based care [8,9,10]. Comprehensive geriatric assessment (CGA) is usually a cornerstone of hospital-at-home services tailored to older people. Its implementation in this setting is cost-effective and has been associated with similar outcomes as hospital admission and reduced risk of long-term residential care admission in the 6-month period following service activation [8,12,13]. The ideal beneficiaries of these services should be medically unwell but physiologically stable, older patients with a sufficient level of home assistance by non-specialized caregivers [12,13].

The hospital-at-home programs described in the existing literature do not always involve specialist diagnostic examinations delivered at the patient’s own home during the acute phase of illness since, in many cases, the activation of the service is made after an ED visit, where laboratory and instrumental tests have already been performed [9,10,11]. Thus, many of these services were designed to avoid hospital admissions and not ED visits.

In our academic hospital, we have implemented a domiciliary care service specifically tailored to older patients, the Multidisciplinary Mobile Unit (MMU) [14]. This service gives emphasis to a multidisciplinary home assessment of older patients with urgent complaints, providing diagnostic and prognostic evaluations not only with traditional semeiotics, comprehensive geriatric assessment (CGA), and specialist consult but also with the aid of portable technologic equipment, including ultrasound, spirometry, and point-of-care lab tests such as blood gas analysis [14]. This hospital-at-home model was originally developed for nursing home residents in 2018–2019 [14] and then was extended to patients living in the community during the coronavirus disease-19 (COVID-19) pandemic [15].

The objective of this study was to assess the feasibility of the MMU model by describing the complexity of the case mix of older patients who were evaluated over an eight-month period, assessing the rate of hospital referral immediately after MMU visit and after 30-day follow-up, and mortality. A secondary aim was also to evaluate the degree of satisfaction of patients and their caregivers with the care received during UMM visits.

## 2. Materials and Methods

### 2.1. Study Setting and Population

This observational prospective study was conducted in January–August 2023 within the MMU service, activated in the Geriatric-Rehabilitation Department of Parma University Hospital. This hospital has around 1000 acute-care beds and serves both urban and rural areas of Parma province, Emilia-Romagna region, Italy, with an overall catchment area of around 300,000 people.

We considered for inclusion all patients who were visited at home by the MMU for the first time upon request from the general practitioner (GP) or an outpatient specialist service. Patients who agreed to participate and signed informed consent were finally included. The presence of an available caregiver willing to be contacted by phone for follow-up reasons was an additional inclusion criterion. Conversely, we excluded from the study all patients who were already followed up by the MMU at the moment of study start, physiologically unstable patients who required immediate hospitalization, and subjects unable to provide informed consent.

### 2.2. Outline of the MMU Service

The characteristics of the MMU before and during the COVID-19 pandemic have already been detailed elsewhere [14,15]. This service, originally intended only for nursing home residents needing specialist consultation, has been extended to home visits in late 2020 with the emergence of the second and third wave of the COVID-19 pandemic, providing diagnostic evaluation and treatment mainly of patients with SARS-CoV-2 infection. Then, with the evolution of the pandemic, the MMU service shifted to non-COVID-19 patient care, especially for older subjects with acute medical problems and stable vital signs. In this case, the service was activated by the GP or other community specialists after they had completed their first-level assessment and in case of uncertainty on hospital referral for further examinations or admission. This is the organization of the MMU in force while the study presented here was performed. 

The MMU team is composed of hospital physicians with specialization in different fields of internal medicine (including gastroenterology, pneumology, and clinical nutrition), sharing certification in clinical ultrasonography and experience in geriatric care. The composition of the team is chosen on a case-by-case basis among the staff of the Geriatric-Rehabilitation Department, depending on the reasons leading to service activation. The activation of MMU is requested by phone during workdays from 8 a.m. to 8 p.m. The GP or community specialist responsible for the activation presents the clinical case to the physician in charge of the MMU team. The MMU physician can then simply provide remote consultancy or arrange a home visit. In this case, a team of at least two specialists reaches the patient’s home and provides consultation on-site. The team is equipped with a portable ultrasound system, spirometer, and point-of-care lab diagnostics. Urgent first-level invasive procedures, including vascular access positioning, bladder catheter, and nasogastric tube insertion, can be performed during the visit. The team can also administer urgent intravenous treatments, such as loop diuretics, steroids, fluids, and antibiotics, facilitating the activation of home nursing care services for subsequent administrations.

Geriatric multi-site ultrasound is a cornerstone of the MMU consultation [16,17]. In many situations, the integration of clinical data with ultrasound findings allows one to better focus the diagnosis and quickly initiate an appropriate treatment. This is particularly true for respiratory diseases, where ultrasound, in the hands of expert operators, has diagnostic capacity in many cases similar to chest computed tomography [16,17].

Based on the multidisciplinary assessment, the MMU team can provide personalized advice and referrals to each patient, discussing the care plan with the GP or community specialist responsible for the call. If stabilization on site is not possible, the MMU can plan direct hospital admission, avoiding ED access. Otherwise, the MMU can provide a follow-up schedule, also with the activation of home nursing services, and plan further home visits for monitoring the clinical evolution.

### 2.3. Study Procedures and Ethical Statement

After obtaining an informed consent signature, data were collected from MMU clinical records on an electronic database. These data included the patient’s chief complaint, the timing of the visit, the diagnostic hypothesis of the physician responsible for the activation, the number and type of chronic comorbidities, the level of functional performance of the patient, drug treatments, and the type of social network supporting patient care.

Comorbidities were measured through the calculation of the Cumulative Illness Rating Scale (CIRS), Comorbidity Score (CIRS-CS), and Severity Index (CIRS-SI). CIRS-CS was calculated as the sum of the ranks between 0 and 4 assigned to each of the 14 items representing the main organs, systems, or anatomical districts potentially affected by chronic illness, while CIRS-SI was determined as the number of items with a score of 3 and 4 [18]. The presence of more than one chronic condition in the same organ, district, or anatomical district was considered to assign a higher score than the sum of the intrinsic severity of each condition to the corresponding item of CIRS. 

Frailty was measured with the Clinical Frailty Scale (CFS) in accordance with the deficit accumulation model proposed by Rockwood and colleagues [19]. The CFS is a 9-point scale measuring the level of fitness and functional performance, both in the physical and cognitive domain, of older individuals. It is based on routine clinical and anamnestic data collected during the first approach to geriatric patients and physical examination. Patients are defined as frail if their CFS is >4.

The type of diagnostic procedures delivered by the MMU team and the therapies administered were also collected in the dataset. The outcome of the MMU consultation (hospital referral vs. home care) was considered as the primary study endpoint. Hospital admission and mortality 30 days after the MMU visit, assessed through a phone call with the patient or his/her caregiver, were considered secondary endpoints. 

During the MMU visit, the team also administered to the patient and caregivers a customer satisfaction questionnaire based on the GSQ-8 (General Satisfaction Questionnaire-8) [20] in order to investigate the level of satisfaction for the human interaction with MMU personnel and care received. The MMU team was blind to the results of the questionnaire.

The study protocol was approved by the competent Ethics Committee under the ID 790/2022/OSS/AOUPR, approval date: 20 December 2022. All the study procedures were conducted in accordance with the Declaration of Helsinki.

### 2.4. Statistical Analyses

Variables were expressed as median and interquartile range (IQR) or percentages, as appropriate. The demographic, anamnestic, and clinical characteristics of participants were compared between groups of participants categorized according to the type of MMU activation (GP vs. outpatient specialist), outcome of the consultation (hospital referral vs. ongoing home care), and outcome of follow-up (hospitalization vs. no-hospitalization after 30 days). These comparisons were made with Mann–Whitney, Fisher exact, or Chi-square tests. The factors significantly and independently associated with hospitalization after MMU visit, hospitalization, and mortality on 30-day follow-up were identified with stepwise binary logistic regression models. The output of GSQ-8 questionnaires was analyzed in descriptive form. The SPSS package (v.28, IBM, Armonk, NY, USA) was used for analyses. *p*-values were considered significant when <0.05.

## 3. Results

**Figure 1:**
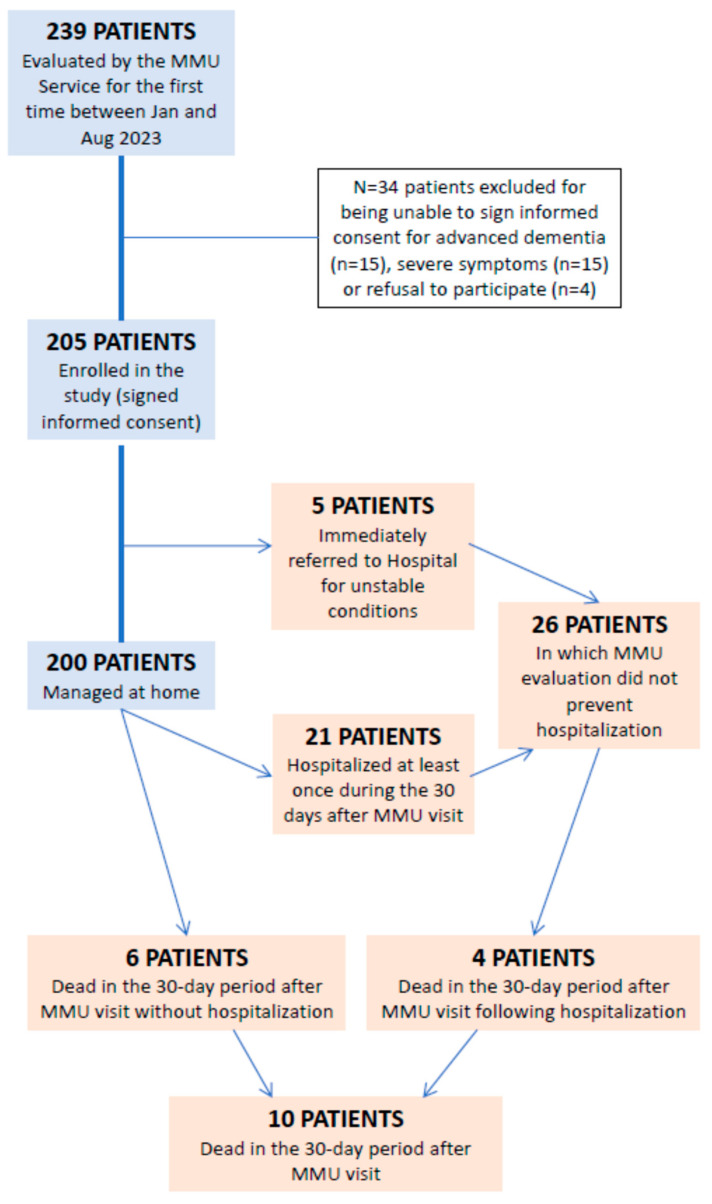
Flowchart of study participants.

The study flow chart is depicted in Figure 1. Among the 205 participants (102 men, age median 83, IQR 76–88 years old), the most frequent chief complaints justifying MMU activation were dyspnea (49%), cough (34%), musculoskeletal pain (17%), rapid decline of functional performance (17%), peripheral edema (15%) and fever (9%). COPD exacerbation (24%), bacterial pneumonia (16%), acute heart failure (14%), and COVID-19 infection (9%) were the most frequent diagnostic hypotheses formulated by physicians responsible for MMU activation. Participants had high levels of multimorbidity (median CIRS-CS 11, IQR 7–14; median CIRS-SI 2, IQR 1–3), polypharmacy (median number of drugs 5, IQR 3–8) and frailty (median CFS 6, IQR 4–7) (Table 1). The prevalence of frailty (CFS > 4) was 72%. The most frequent diagnostic test provided by the MMU team was thoracic ultrasound (81%), followed by abdominal ultrasound (18%) and arterial blood gas analysis (9%). The most frequent diagnoses made after MMU evaluation were COPD exacerbation (22%), bacterial pneumonia (15%), and acute heart failure (13%). The most frequent pharmacologic prescriptions of the MMU team were antibiotics (19%), steroids (11%), and diuretics (8%).

Only 5 patients out of 205 (2.4%) were hospitalized as a result of the MMU evaluation. An overview of their characteristics is provided in Table 2. These patients had, on average, a higher burden of comorbidities (median CIRS-CS 18, IQR 14–23, vs. 11, IQR 7–14, *p* = 0.010) and worse respiratory exchanges (median peripheral O2 saturation 93, IQR 88–97, vs. 97, IQR 95–98, *p* = 0.039).

After a 30-day follow-up period, 21 patients (10.2%) were hospitalized and 10 patients (4.9%) were dead. A comparison of the characteristics of participants who were hospitalized at follow-up and those who were not is provided in Table 3. Table 4 provides a comparison of the characteristics of patients dead at follow-up versus survivors.

In a stepwise binary logistic regression model, the factors significantly associated with hospital admission one month after the MMU visit were age, cancer, and abdominal pain (Table 5). The factors significantly associated with death in a stepwise binary logistic regression model were loss of mobility as the main reason for MMU activation and CIRS-SI (Table 5). Thirty-day mortality was also higher in patients referred to MMU service by GPs, in comparison with patients referred by other specialists, despite a lower prevalence of frailty (Table 6).

The GSQ-8 questionnaire revealed a high degree of satisfaction of patients and caregivers for the care received during the MMU home access (Table 7).

## 4. Discussion

Our data suggest that the MMU model is a feasible and effective strategy to manage urgent clinical complaints of older patients directly at their own homes, especially related to respiratory illness, avoiding hospital referral for diagnostic or treatment purposes. Point-of-care ultrasonography, in particular, seems to be of paramount importance for facilitating the diagnostic process outside the hospital [16,17]. Thoracic ultrasound has proven diagnostic effectiveness, particularly in older frail patients, where traditional first-level exams, such as chest X-ray, may lack sensitivity [21]. The use of thoracic ultrasound for ruling in or ruling out the most common causes of dyspnea can also significantly shorten the diagnostic time and improve patient care [22]. The implementation of ultrasound diagnostics in the community, especially in settings of geriatric care, may substantially contribute to reducing unnecessary hospitalizations and avoiding hospital-related complications in older patients [16]. Ultrasound, however, is highly dependent on the examiner’s skills and experience, and its role and effectiveness in patient outcomes remain debated in the absence of rigorous randomized clinical trials and studies focused on cost-effectiveness [23]. 

In the last decade, interventions in the field of geriatric urgency have been mainly focused on delivering CGA to older patients in the community or at the hospital front door in order to intercept frailty and improve care management and disposition accordingly [13,24,25]. Only a limited number of organizational interventions centered on the needs of older people consisted of bringing the hospital proficiency directly to the patient’s own home [8,9,10,11,12,13]. In most of the existing literature, hospital-at-home programs are, in fact, activated after a first diagnostic evaluation of the patient in an Emergency Department or other acute-care facility [8,9,10,11,12,13]. So, the interventions delivered at the patient’s home are generally limited to CGA, pharmacological treatment, and periodical monitoring [8,9,10,11,12,13].

During the COVID-19 pandemic peaks, however, mobile units composed of hospital specialists and nurses were activated in some areas in order to deliver diagnostics and therapeutic advice directly in territorial health facilities [26,27]. These experiences were associated with a high level of efficiency in preventing hospital admissions, and patient outcomes were the same, if not better, than those of patients who were hospitalized for COVID-19. The spirit of the MMU model is the same, even if applied to complex geriatric patients without COVID-19. The aim of the MMU model is, in fact, to provide a complete consultation in an urgent situation, that may be normally achieved only in the hospital context. CGA, of course, can be a part of the MMU evaluation process, but it is embedded into a multidisciplinary evaluation considering also the level of urgency and the priority of reaching on-site stabilization of the patient. The results of our study suggest that this goal can be more difficult to achieve in subjects with severe multimorbidity and cancer. Therefore, specific pathways should be developed for home care of older patients with cancer or severe frailty, considering the principles of palliative care [28,29]. 

Some limitations of our investigation should be considered. First, the observational study design, lacking a control group not receiving the intervention, does not affirm that MMU is effective in preventing hospital admissions. Second, the circumstance that MMU was activated by GPs or community specialists cannot allow to exclude selection bias. In fact, the MMU service was implemented for home consultation during the COVID-19 pandemic peaks. Therefore, GPs may have been more prone to activate the MMU team in case of urgent respiratory symptoms or in situations requiring an urgent ultrasound evaluation rather than in other situations. Interestingly, in MMU encounters referred by GPs, the prevalence of frailty was lower, but 30-day mortality was higher (Table 6), probably reflecting a higher severity of acute conditions in that patients in comparison with those referred by other specialist physicians, who may not have been particularly familiar with age-related frailty and its complications.

Furthermore, the remote and on-site monitoring performed by the MMU team after the first visit was not included in this study, basically because the follow-up schedule is personalized and adapted to each clinical situation. Thus, 30-day phone monitoring was the only follow-up method considered for the present investigation. No data on MMU cost were also collected, and the evaluation of MMU cost-effectiveness was not possible. 

Finally, the number of recorded hospitalization events was very low, which reduced the power of statistical analyses exploring factors associated with the need for immediate hospitalization and 30-day hospital admissions. This circumstance supports the potential effectiveness of the MMU service for preventing hospital admissions but may also represent a limitation of this study, whose aim was, in any case, to assess the feasibility of the model. Larger studies should be designed in the future to specifically assess the impact of the MMU service on patient-centered outcomes, not just in terms of hospital referral but also considering functional performance in the long term.

The MMU model was developed in the context of a mid-size town in Northern Italy, with only one acute-care hospital providing care for around 300,000 inhabitants. The Italian national healthcare system is universal and public and provides free or low-cost care, including GP access, treatment at public hospitals, subsidized drugs, lab services, and specialist care for most disciplines. The reproducibility of the MMU model should account for these particular circumstances because mobile specialist unit services or hospital-at-home programs may have different effectiveness based on the local organization of health care. Therefore, the development of future care interventions inspired by the MMU model should be adapted to the specific needs of each organizational context and consider the issue of reproducibility in different geographical locations. 

## 5. Conclusions 

The MMU model represents a feasible and promising strategy to manage urgent clinical complaints of older patients directly at their own homes, with a high potential of limiting ED visits and hospital admissions. Extreme ages, the presence of cancer, and abdominal pain were associated with a higher risk of hospitalization, while high levels of functional dependence and disease severity were associated with 30-day mortality. Future studies should assess the impact of complex interventions tailored to older patients, like the MMU, on the utilization of healthcare resources, costs, and patient-centered outcomes. 

## Figures and Tables

**Table 1:** Overview of the main characteristics of the studied population (n = 205 subjects visited at home by the MMU service) and of the interventions delivered by the MMU.

Parameter	Median (IQR) or Percentage
***General characteristics of patients***	
Age, years	83 (76–88)
Females, %	50
COPD, %	43
Heart failure, %	52
Diabetes, %	25
Chronic kidney disease, %	21
Dementia, %	18
Cancer, %	14
CIRS cardiac subscore	2 (1–3)
CIRS hypertension subscore	2 (0–3)
CIRS blood and vessel subscore	0 (0–1)
CIRS respiratory subscore	3 (1–3)
CIRS head and neck	0 (0–0)
CIRS superior gastrointestinal	0 (0–1)
CIRS inferior gastrointestinal	0 (0–1)
CIRS liver	0 (0–0)
CIRS kidney	0 (0–2)
CIRS urinary tract	0 (0–1)
CIRS musculoskeletal and skin	1 (0–2)
CIRS nervous system	0 (0–1)
CIRS endocrine	0 (0–1)
CIRS psychiatric	0 (0–0)
CIRS-CS	11 (7–14)
CIRS-SI	2 (1–3)
CFS	6 (4–7)
Drugs, number	5 (3–8)
Living alone, %	15
***Mode of MMU activation***	
MMU activated by the GP, %	53
MMU activated by another specialist, %	47
***Cause of MMU activation/Patient complaints***	
Dyspnea, %	49
Cough, %	34
Musculoskeletal pain, %	17
Loss of mobility, %	17
Peripheral edema, %	15
Fever, %	9
Abdominal pain, %	7
***Diagnostic procedures delivered by the MMU***	
Thoracic ultrasound, %	81
Peripheral saturation monitoring, %	75
Abdominal ultrasound, %	18
Arterial blood gas analysis, %	9
Echocardiography, %	6
***Final diagnosis made by the MMU evaluation***	
COPD exacerbation, %	22
Bacterial pneumonia, %	15
Acute heart failure, %	13
COVID-19, %	8
Other, %	42
***Most frequent pharmacological prescriptions by the MMU***	
Antibiotics, %	19
Corticosteroids, %	11
Diuretics, %	8
Oxygen therapy, %	4

MMU = Multidisciplinary Mobile Unit; COPD = Chronic Obstructive Pulmonary Disease; CIRS-CS = Cumulative Illness Rating Scale-Comorbidity Score; CIRS-SI = Cumulative Illness Rating Scale-Severity Index; CFS = Clinical Frailty Scale; COVID-19 = Coronavirus Disease-19.

**Table 2:** Comparison of the main characteristics of patients who were hospitalized immediately after MMU visit and those who were managed at home.

Parameter	Patients Managed at Home after Initial Evaluation (N = 200)	Patients Referred to Hospitalization (N = 5)	*p*
Age, years	83 (76–88)	85 (74–94)	0.511
Females, %	51	0	0.059
COPD, %	43	60	0.654
Heart failure, %	51	80	0.371
Diabetes, %	24	60	0.096
Chronic kidney disease, %	20	60	0.060
Dementia, %	19	0	0.587
Cancer, %	14	0	1.000
CIRS-CS	11 (7–14)	18 (14–23)	**0.010**
CIRS-SI	2 (1–3)	5 (3–6)	**0.004**
CFS	6 (4–7)	6 (5–8)	0.531
Drugs, number	5 (3–8)	8 (4–10)	0.188
Living alone, %	15	20	0.563
MMU activated by the GP, %	53	80	0.376
MMU activation for dyspnea, %	49	60	0.680
MMU activation for cough, %	34	40	1.000
MMU activation for musculoskeletal pain, %	17	20	1.000
MMU activation for loss of mobility, %	17	20	1.000
MMU activation for peripheral edema, %	15	20	0.550
MMU activation for fever, %	10	0	1.000
MMU activation for abdominal pain,%	7	0	1.000
Peripheral O_2_ saturation during visit	97 (95–98)	93 (88–97)	**0.039**
Respiratory frequency during visit	14 (14–14)	16 (15–18)	**0.011**
Thoracic ultrasound during MMU visit, %	80	100	0.585
Abdominal ultrasound during MMU visit, %	17	40	0.217
Final diagnosis: COPD exacerbation, %	22	20	1.000
Final diagnosis: acute heart failure, %	13	20	0.518
Final diagnosis: bacterial pneumonia, %	15	20	0.571
Final diagnosis: COVID-19	8	0	1.000

MMU = Multidisciplinary Mobile Unit; COPD = Chronic Obstructive Pulmonary Disease; CIRS-CS = Cumulative Illness Rating Scale-Comorbidity Score; CIRS-SI = Cumulative Illness Rating Scale-Severity Index; CFS = Clinical Frailty Scale; GP = General Practitioner; COVID-19 = Coronavirus Disease-19. Data are shown as median and interquartile ranges or percentages. *p*-values calculated with Mann–Whitney or Fisher exact test. *p* < 0.05 are indicated in bold.

**Table 3:** Comparison of the main characteristics of patients who were hospitalized at follow-up (30 days after MMU visit) and those who were not.

Parameter	Patients Not Hospitalized on Follow-Up (N = 179)	Patients Hospitalized on Follow-Up (N = 21)	*p*
Age, years	82 (76–88)	86 (83–93)	**0.006**
Females, %	47	62	0.209
COPD, %	45	33	0.295
Heart failure, %	51	57	0.570
Diabetes, %	24	24	0.998
Chronic kidney disease, %	22	19	1.000
Dementia, %	18	19	1.000
Cancer, %	12	29	**0.043**
CIRS-CS	11 (7–14)	11 (7–14)	0.952
CIRS-SI	2 (1–3)	2 (0–4)	0.875
CFS	6 (4–7)	6 (5–7)	0.356
Drugs, number	5 (3–8)	5 (2–8)	0.801
Living alone, %	14	14	1.000
MMU activated by the GP, %	53	52	0.945
MMU activation for dyspnea, %	48	38	0.392
MMU activation for cough, %	34	24	0.343
MMU activation for musculoskeletal pain, %	17	29	0.226
MMU activation for loss of mobility, %	16	29	0.219
MMU activation for peripheral edema, %	15	19	0.748
MMU activation for fever, %	10	10	1.000
MMU activation for abdominal pain, %	5	24	**0.010**
Peripheral O_2_ saturation during visit	97 (95–98)	97 (94–98)	0.442
Respiratory frequency during visit	14 (14–15)	14 (14–14)	0.097
Thoracic ultrasound during MMU visit, %	79	81	1.000
Abdominal ultrasound during MMU visit, %	17	33	0.081
Final diagnosis: COPD exacerbation, %	22	24	1.000
Final diagnosis: acute heart failure, %	14	5	0.320
Final diagnosis: bacterial pneumonia, %	14	24	0.328
Final diagnosis: COVID-19	8	5	1.000

MMU = Multidisciplinary Mobile Unit; COPD = Chronic Obstructive Pulmonary Disease; CIRS-CS = Cumulative Illness Rating Scale-Comorbidity Score; CIRS-SI = Cumulative Illness Rating Scale-Severity Index; CFS = Clinical Frailty Scale; GP = General Practitioner; COVID-19 = Coronavirus Disease-19. Data shown as median and interquartile range or percentage. *p* values calculated with Mann–Whitney for continuous variables, for dichotomous variables, Chi-square test, or Fisher’s exact test, the latter in the presence of items with expected value less than 5. *p* < 0.05 are indicated in bold.

**Table 4:** Comparison of the main characteristics of patients who were dead on follow-up (30 days after MMU evaluation) and those who were not.

Parameter	Survivors on Follow-Up (N = 195)	Dead on Follow-Up (N = 10)	*p*
Age, years	82 (76–88)	92 (85–93)	**0.010**
Females, %	49	50	1.000
COPD, %	45	20	0.190
Heart failure, %	52	50	1.000
Diabetes, %	24	20	1.000
Chronic kidney disease, %	22	20	1.000
Dementia, %	17	40	0.083
Cancer, %	13	30	0.135
CIRS-CS	11 (7–14)	13 (7–17)	0.469
CIRS-SI	2 (1–3)	4 (3–5)	**0.001**
CFS	6 (4–7)	7 (4–8)	0.185
Drugs, number	5 (3–8)	4 (2–8)	0.687
Living alone, %	14	10	1.000
MMU activated by the GP, %	51	90	**0.020**
MMU activation for dyspnea, %	48	40	0.752
MMU activation for cough, %	35	10	0.169
MMU activation for musculoskeletal pain, %	18	10	0.694
MMU activation for loss of mobility, %	16	40	0.075
MMU activation for peripheral edema, %	15	20	0.654
MMU activation for fever, %	10	10	1.000
MMU activation for abdominal pain, %	8	0	1.000
Peripheral O_2_ saturation during visit	97 (95–98)	97 (94–98)	0.263
Respiratory frequency during visit	14 (14–15)	14 (14–15)	0.988
Thoracic ultrasound during MMU visit, %	79	100	0.208
Abdominal ultrasound during MMU visit, %	18	33	0.373
Final diagnosis: COPD exacerbation, %	23	11	0.687
Final diagnosis: acute heart failure, %	13	22	0.332
Final diagnosis: bacterial pneumonia, %	15	22	0.633
Final diagnosis: COVID-19	7	11	0.502

MMU = Multidisciplinary Mobile Unit; COPD = Chronic Obstructive Pulmonary Disease; CIRS-CS = Cumulative Illness Rating Scale-Comorbidity Score; CIRS-SI = Cumulative Illness Rating Scale-Severity Index; CFS = Clinical Frailty Scale; GP = General Practitioner; COVID-19 = Coronavirus Disease-19. Data shown as median and interquartile range or percentage. *p*-values calculated with the Mann–Whitney or Fisher exact test. *p* < 0.05 are indicated in bold.

**Table 5:** Factors associated with hospitalization 30 days after the MMU visit (model 1) and with mortality 30 days after the MMU visit (model 2) in stepwise logistic regression analysis.

Parameter	OR (95% CI)	*p*
***Model 1: Factors associated with hospital admission 30 days after MMU visit***		
Age, years	1.103 (1.029–1.182)	**0.006**
Cancer	4.021 (1.229–13.150)	**0.021**
MMU activation for abdominal pain	9.439 (2.348–37.953)	**0.002**
***Model 2: Factors associated with mortality 30 days after MMU visit***		
MMU activation for loss of mobility	4.257 (1.021–17.739)	**0.047**
CIRS-SI	1.688 (1.222–2.332)	**0.002**

OR = Odds Ratio; CI = Confidence Interval; MMU = Multidisciplinary Mobile Unit; CIRS-SI = Cumulative Illness Rating Scale-Severity Index. *p* < 0.05 are indicated in bold. Entries for model 1: age, cancer, MMU activation for abdominal pain, respiratory frequency (variables with *p* < 0.1 in Table 3). Entries for model 2: age, CIRS-SI, dementia, MMU activation for loss of mobility (variables with *p* < 0.01 in Table 4).

**Table 6:** Comparison of the prevalence of frailty and patient outcomes after categorization of study participants by pathway of MMU referral (activation by GP or another specialist).

Parameter	MMU Activated by GP (N = 110)	MMU Activated by Other Specialist (N = 95)	*p*
Frailty (CFS > 4), %	65	80	**0.018**
Hospitalization immediately after MMU visit, %	3.6	1.1	0.376
Hospitalization within 30 days from MMU visit, %	11	11	0.974
Mortality 30 days after MMU visit, %	8.7	1.1	**0.020**

Data are shown as percentages. *p*-values are calculated with the Fisher exact test or Chi-square test. *p* < 0.05 are indicated in bold.

**Table 7:** Overview of the overall results of the GSQ-8 questionnaire on customer satisfaction provided by the 205 patients enrolled in the study or their caregivers.

	Very Satisfied—Definitely Yes	Satisfied—Quite Yes	Not Much Satisfied—Quite No	Very Unsatisfied—Definitely No
**Question 1**	91.2%	8.8%	0%	0%
**Question 2**	93.7%	5.4%	0.5%	0.5%
**Question 3**	96.1%	3.9%	0%	0%
**Question 4**	97.1%	2.9%	0%	0%
**Question 5**	97.1%	2.9%	0%	0%
**Question 6**	91.2%	8.3%	0%	0.5%
**Question 7**	95.6%	4.4%	0%	0%
**Question 8**	92.7%	6.8%	0.5%	0%

Question 1: Did you get a good impression from the reception once you met the medical staff? Question 2: Did you receive the service you expected? Question 3: If a friend of yours was in need of medical help, would you recommend to address to this service? Question 4: Are you satisfied with the care and help received from the medical staff? Question 5: If you were in the same situation of needing a medical consultation, would you call this service again? Question 6: Are you overall satisfied with the ensemble of medical care provided by this service? Question 7: How much are you satisfied with the service working as a team? Question 8: Did the service satisfy your need of medical care?

## Data Availability

The dataset is available in anonymous form upon reasonable request to the corresponding author and to the data controller (Azienda Ospedaliero-Universitaria di Parma).
